# Supplementary material for: Gender discrimination in Swedish family courts: A quantitative vignette study
Source: PLoS One. 2024 Aug 27;19(8):e0296210. doi: 10.1371/journal.pone.0296210 (PMC11349174; doi:10.1371/journal.pone.0296210)
Supplement: S1 File — (PDF) [file pone.0296210.s001.pdf]

Riskbedömning i vårdnadsutredningar.

Högskolan i Gävle

Studien följer Vetenskapsrådets etiska föreskrifter och allmänna råd vid forskning. Medverkande i studien garanteras anonymitet. Vid frågor kan ni kontakta Jukka Kaski vsn08jki@student.hig.se eller Elin Tegnér hco08etr@student.hig.se

Antal år i tjänsten:

Yrkestitel:

Man/kvinna:

Ålder:

Studien undersöker vårdnadsutredares riskbedömning, av fysiskt våld, försummelse och bortförande av barn i en tvist mellan föräldrar. Med fysiskt våld menar vi slag, sparkar, knuffar och/eller att barnet utsätts för våld på andra liknande sätt som uppfattas nedvärderande med uppsåt att skada barnet. Med försummelse menar vi psykisk och fysisk försummelse. I psykisk försummelse ingår känslomässig tillgänglighet, trygghet, kärlek och att barnet får sina behov uppfyllda. I fysisk försummelse menar vi att barnet får mat, kläder, motion, en ren och bekväm säng och att barnet får tillgång till sjukvård och tandläkarvård. Med bortförande menar vi att den ena föräldern olovligen för bort barnet från den andre föräldern och på det viset försvårar dennes möjlighet att ha en relation med barnet.

Idag: Peter och Ulla bor isär sedan sex månader i Eskilstuna. De har en dotter Emma, som är sex år gammal. Peter bor idag i en lägenhet som består av två rum och kök. Ulla har i sin tur köpt en liten stuga med tre rum och kök i utkanten av orten. Emma bor varannan vecka hos Peter och Ulla med delad vårdnad.

Peters bakgrund: Peter kommer ursprungligen från Norrbotten, han har växt upp i ett hem där det har förekommit alkohol och droger, själv varken röker eller dricker Peter. Hans föräldrar är skilda och han har dålig kontakt med sin far. Han träffade Ulla på en Finlandskryssning och de blev då ett par. Peter flyttade ner till Ulla och de fick en dotter tillsammans. Peter är snickare och oftast arbetar han 12-14 timmar om dagen. Peter har en bra relation till dottern, men det är Ulla som tagit huvudansvaret gällande kvartssamtal och föräldramöten. Detta har varit en överenskommelse föräldrarna emellan med hänsyn till Peters arbetssituation och deras gemensamma ekonomi. Peter och Ulla har inte alltid varit överens om hur man uppfostrar barn, Peter har ibland sagt att han tycker att Ulla är för vek i sin uppfostran. Ulla i sin tur tycker att Peter är för hårdhänt och tror att han har luggat Emma flera gånger. Peter förnekar dock detta. Peter har få närboende vänner och har inte riktigt känt sig hemma på orten. Emma har ingen kontakt med sina farföräldrar. För fem år sedan fick Peter veta att hans mor drabbats av cancer, han gick då in i en depression. Idag mår Peter bättre men äter fortfarande antidepressiva mediciner och går sporadiskt i samtal hos psykolog.

Ulla och hennes bakgrund: Ulla är uppvuxen i Eskilstuna, hon är utbildad sjuksköterska och arbetar på en vårdcentral. Ulla är uppvuxen tillsammans med sin yngre syster och sina föräldrar. Ulla arbetar kontorstider vilket har varit en förutsättning för att det ska fungera med Emma eftersom Peter har så långa arbetsdagar. Ulla har haft en trygg barndom och har ett brett nätverk på orten bestående av vänner och nära släkt. Emma har god kontakt med sina morföräldrar som passat henne flera gånger.

Separationen: Peter har berättat att det kom som en överraskning för honom att Ulla ville skiljas, visst hade de problem men han trodde att de skulle gå att lösa. Peter tror att separationen beror på att Ulla träffat någon annan, Ulla i sin tur berättar att Peter inte tog ansvar för vare sig Emma eller sig själv och att hon inte orkade med det längre. Separationen har varit konfliktfylld, Peter har sagt att hans barn inte ska bli ett ”skilsmässobarn” och han beskyller Ulla för att förstöra familjen. Sedan separationen har Peter och Ulla haft stora svårigheter att samarbeta kring Emma, de kan inte ens komma överens om i vilken skola hon ska gå. Peter har vid upprepade tillfällen hotat att han ska flytta till norrbotten och ta Emma med sig. Peter vill vara en god far och han är mån om Emma, Fem gånger senaste månaden har Peter dock glömt bort att hämta Emma på fritids. Ullas föräldrar har då hämtat Emma. Ulla har tagit upp detta och Peter har lovat att det inte ska hända igen. Emma har också uttryckt att hon vill vara hemma hos modern. Ulla har slutligen tröttnat och begär nu ensam vårdnad om Emma, Peter vill fortsätta ha delad vårdnad och boende.

Vi är intresserade av att ta del hur Du som handläggare bedömer risknivån som kan finnas kring Peter och hans relation till Emma.

Riskbedömning

Besvara frågorna med ett kryss där Du anser att riskerna ligger.

Fråga ett: Anta att Peter och Ulla tilldelas delad vårdnad och boende för Emma. Hur stor risk anser Du att det är att Peter kommer att försumma Emma fysiskt?

Minimal Risk____ Max Risk

Fråga två: Anta att Peter och Ulla tilldelas delad vårdnad och boende för Emma. Hur stor risk anser Du att det är att Peter kommer att försumma Emma psykiskt?

Minimal Risk____Max Risk

Fråga tre: Hur stor är risk tror Du att det är att Peter kommer att föra bort Emma från modern?
Minimal Risk____ Max Risk

Fråga fyra: Hur stor risk tror Du att det är att Peter kommer att utsätta Emma för fysiskt våld?
Minimal Risk____Max Risk

<u>Utredning för barnets bästa</u>

Anta att du är utredare i ärendet och ska genomföra en riskbedömning utifrån den information som är presenterad ovan. Ditt uppdrag är att se och göra en bedömning av om Peter och Ulla ska ha delad vårdnad om Emma, alternativt om de ska ha gemensam vårdnad. Du får också möjlighet att bedöma i umgängesfrågan om det känns mer aktuellt. Hur vill Du rekommendera i ärendet? Sätt en ring kring det Du anser vara lämpligt.

1: Ulla får enskild vårdnad och Peter ska ha begränsat umgänge

2: Ulla får enskild vårdnad och Peter ska ha omfattande umgänge.

3: Gemensam vårdnad med lika stort umgänge.

4: Gemensam vårdnad med stadigvarande boende hos Ulla, umgänge med Peter varannan helg.

Givet berättelsen ovan, saknades någon speciell detalj i ärendet för att du ska kunna göra en bättre rekommendation?

____

Riskbedömning i vårdnadsutredningar

Högskolan i Gävle

Studien följer Vetenskapsrådets etiska föreskrifter och allmänna råd vid forskning. Medverkande i studien garanteras anonymitet. Vid frågor kan ni kontakta vsn08jki@student.hig.se eller hco08etr@student.hig.se

Antal år i tjänsten:

Yrkestitel:

Man/kvinna:

Ålder:

Studien undersöker vårdnadsutredares riskbedömning, av fysiskt våld försummelse och bortförande av barn i en tvist mellan föräldrar. Med fysiskt våld menar vi slag, sparkar, knuffar och/eller att barnet utsätts för våld på andra liknande sätt som uppfattas nedvärderande med uppsåt att skada barnet. Med försummelse menar vi psykisk och fysisk försummelse. I psykisk försummelse ingår känslomässig tillgänglighet, trygghet, kärlek och att barnet får sina behov uppfyllda. I fysisk försummelse menar vi att barnet får mat, kläder, motion, en ren och bekväm säng och att barnet får tillgång till sjukvård och tandläkarvård. Med bortförande menar vi att den ena föräldern olovligen för bort barnet från den andre föräldern och på det viset försvårar dennes möjlighet att ha en relation med barnet.

Idag: Ulla och Peter bor isär sedan sex månader i Eskilstuna. De har en dotter Emma, som är sex år gammal. Ulla bor idag i en lägenhet som består av två rum och kök. Peter har i sin tur köpt en liten stuga med tre rum och kök i utkanten av orten. Emma bor varannan vecka hos Ulla och Peter med delad vårdnad.

Ullas bakgrund: Ulla kommer ursprungligen från Norrbotten, hon har växt upp i ett hem där det har förekommit alkohol och droger, själv varken röker eller dricker Ulla. Hennes föräldrar är skilda och hon har dålig kontakt med sin far. Hon träffade Peter på en Finlandskryssning och de blev då ett par. Ulla flyttade ner till Peter och de fick en dotter tillsammans. Ulla är vårdbiträde på ortens ålderdomshem, oftast arbetar hon 12-14 timmar om dagen. Ulla har en bra relation till dottern, men det är Peter som tagit huvudansvaret gällande kvartssamtal och föräldramöten. Detta har varit en överrenskommelse föräldrarna emellan med hänsyn till Ullas arbetssituation och deras gemensamma ekonomi. Ulla och Peter har inte alltid varit överens om hur man uppfostrar barn, Ulla har ibland sagt att hon tycker att Peter är för vek i sin uppfostran. Peter i sin tur tycker att Ulla är för hårdhänt och tror att hon har luggat Emma flera gånger. Ulla förnekar dock detta. Ulla har få närboende vänner och har inte riktigt känt sig hemma på orten. Emma har ingen kontakt med sina morföräldrar. För fem år sedan fick Ulla veta att hennes mor drabbats av cancer, hon gick då in i en depression. Idag mår Ulla bättre men äter fortfarande antidepressiva mediciner och går sporadiskt i samtal hos psykolog.

Peters bakgrund: Peter är uppvuxen på den orten där han bor idag, han är utbildad snickare och arbetar på sin fars företag. Peter är uppvuxen tillsammans med sin yngre syster och sina föräldrar. Peter arbetar kontorstider vilket har varit en förutsättning för att det ska fungera med Emma eftersom Ulla har så långa arbetsdagar. Peter har haft en trygg barndom och har ett brett nätverk på orten bestående av vänner och nära släkt. Emma har god kontakt med sina morföräldrar som passat henne flera gånger.

Separationen: Ulla har berättat att det kom som en överraskning för henne att Peter ville skiljas, visst hade de problem men hon trodde att de skulle gå att lösa. Ulla tror att separationen beror på att Peter träffat någon annan, Peter i sin tur berättar att Ulla inte tog ansvar för vare sig Emma eller sig själv och att han inte orkade med det längre. Separationen har varit konfliktfylld, Ulla har sagt att hennes barn inte ska bli ett ”skilsmässobarn” och hon beskyller Peter för att förstöra familjen. Sedan separationen har Ulla och Peter haft stora svårigheter att samarbeta kring Emma, de kan inte ens komma överens om i vilken skola hon ska gå. Ulla har också vid upprepade tillfällen hotat att hon ska flytta till norrbotten och ta Emma med sig. Ulla vill vara en god mor och hon är mån om Emma, fem gånger senaste månaden har Ulla dock glömt bort att hämta Emma på fritids. Peters föräldrar har då hämtat Emma. Peter har tagit upp detta och Ulla har lovat att det inte ska hända igen. Emma har också uttryckt att hon vill vara hemma hos fadern.

Peter har slutligen tröttnat och begär nu ensam vårdnad om Emma, Ulla vill fortsätta ha delad vårdnad och boende.

Vi är intresserade av att ta del hur Du som handläggare bedömer risknivån som kan finnas kring Ulla och hennes relation till Emma.

<u>Riskbedömning</u>

Besvara frågorna med ett kryss där Du anser att riskerna ligger.

Fråga ett: Anta att Ulla och Peter tilldelas delad vårdnad och boende för Emma. Hur stor risk anser Du att det är att Ulla kommer att försumma Emma fysiskt?

Minimal Risk____ Max Risk

Fråga två: Anta att Ulla och Peter tilldelas delad vårdnad och boende för Emma. Hur stor risk anser Du att det är att Ulla kommer att försumma Emma psykiskt?

____

Minimal Risk____Max Risk

Fråga tre: Hur stor är risk tror Du att det är att Ulla kommer att föra bort Emma från fadern?
Minimal Risk____ Max Risk

Fråga fyra: Hur stor risk tror Du att det är att Ulla kommer att utsätta Emma för fysiskt våld?
Minimal Risk____Max Risk

Utredning för barnets bästa

Anta att du är utredare i ärendet och ska genomföra en riskbedömning utifrån den information som är presenterad ovan. Ditt uppdrag är att se och göra en bedömning av om Peter och Ulla ska ha delad vårdnad om Emma, alternativt om de ska ha gemensam vårdnad. Du får också möjlighet att bedöma i umgängesfrågan om det känns mer aktuellt. Hur vill Du rekommendera i ärendet? Sätt en ring kring det Du anser vara lämpligt.

1: Peter får enskild vårdnad och Ulla ska ha begränsat umgänge.

2: Peter får enskild vårdnad och Ulla ska ha omfattande umgänge.

3: Gemensam vårdnad med lika stort umgänge.

4: Gemensam vårdnad med stadigvarande boende hos Peter, umgänge med Ulla varannan helg.

Givet berättelsen ovan, saknades någon speciell detalj i ärendet för att du ska kunna göra en bättre rekommendation?
____
