# Supplementary material for: Gender discrimination in Swedish family courts: A quantitative vignette study
Source: PLoS One. 2024 Aug 27;19(8):e0296210. doi: 10.1371/journal.pone.0296210 (PMC11349174; doi:10.1371/journal.pone.0296210)
Supplement: Attachment — Submitted filename: Response to Reviewers.docx [file pone.0296210.s002.docx]

Rebuttal letter

First, we like to thank all reviewers for your useful comments and suggestions. We believe that your valuable comments have substantially improved the manuscript. With few exceptions, we have revised the manuscript to address your insightful and important comments. In addition, we have consulted a legal expert who graciously has accepted being a co-author to improve the overall quality and, in particular, the manuscript's legal terminology.

Responses to the reviewer's comments are listed below.

Reviewer #1

Introduction

1. Introduction can be structured in a clearer way.

Response: We have revised the introduction and added a more historical perspective on why mothers are favored. Also, we have added a section, along with a more clear explanation gap: Previous research is either (a) old, (b) outside of Sweden, or (c) using professional social workers as participants. The present study is the only study that satisfies all three conditions.

Theoretical framework

The strength of theoretical framework if more recent literature can be incorporated, especially empirical studies on this. It would be even stronger if there is any literature based on a Swedish sample.

Response: We have broadened the literature search and added more recent relevant Swedish research in Kullberg (2005). Although his vignette study is not directly concerning custody disputes, it suggests favoritism for mothers in parenting. However, as much as we would like to add more recent Swedish research in the field, the number of active Swedish researchers interested in custody disputes are very few. The first author know them by name, and none of them are interested in gender discrimination against men. In fact, as far as we know, the only researchers interested in gender discrimination in Swedish child custody are the authors of this manuscript.

Methods section I

Second, the methods section needs to be improved. Although the design and procedure are explained, more detail on the sample and groups are worth stating. What are the group descriptives, were there any group differences? The sample size is very low, which is understandable with a sample of professionals, but what does this mean for the power of the study?

Response: It means that the effect size (i.e., the gender discrimination) needs to be very high for the study to find significant effects. This is indeed the case. We have added group comparisons in the Design and Participants section.

Methods section II

The sample size is very low, which is understandable with a sample of professionals, but what does this mean for the power of the study?

Response: We have added limited power as a listed weakness in the study, as well as discussing that the effect size could be very strong because of the small data set.

Results and discussion section I

I believe most revisions should be done in the results and discussion section. The result section now only shows statistics for the found difference in recommendations of shared living, but there are more options to answer. For completion, it would be more transparent to also show the non-significant results. Especially because the discussion section does try to explain some of the non-findings, but no statistics are given. The result section should also include some descriptive statistics of the answers, as for example in the discussion the scored weakness of the parents is discussed, but no statistics are provided.

Response: We have added the numerical data on custody despite that only six participants in the whole study recommended sole custody in the entire sample. The study did not include any ratings of parental weakness. We have revised the sentence "However, the weaknesses were rated as more serious for fathers than mothers, and fathers were subsequently more excluded from the child to a larger extent compared to mothers." This sentence is confusing and is revised into "However, the identical weaknesses in both parents only had an effect on fathers, as they were recommended to be more excluded from the child's life to a larger extent compared to mothers."

Results and discussion section II

Furthermore, research on bias is important but before you can conclude that there is a bias, it is important to exclude other possible explanations of differences. Now, nothing is stated about any additional analyses trying to find other explanations such as gender of the participant or years of experience. If this is not possible with the sample size, the authors should consider trying to increase the sample size.

Response: As a consequence of adding group data in the design and participants section, there are now statistical analyses on group data that could have been confounding factors such as participant age or years of experience. We wholeheartedly agree that a larger sample size would be preferable. However, given that it is professionals we are trying to recruit, the acceptance rate is quite low. Also, one has to bear in mind that family court units are small and several of the smaller municipalities (of Sweden's 290) are using the same custody evaluators.

Results and discussion section III

In general, the result and discussion sections require more explanation about made choices for the analysis. The method section mentions four answer options, but the structure and text of the result section makes it difficult to follow which answer options are analyzed or taken together. The second paragraph of the discussion was very hard to follow, I believe the first sentence should be “… sole living but not sole custody…” instead of shared custody.

Response: The results are dependent on participants' recommendations. For instance, there is a floor effect, where only six participants recommended sole custody which is insufficient for statistical analysis. We have added a section explaining the intricate relation between custody, living and visitation recommendations to introduce our analysis. We have revised and simplified the wording to increase intelligibility in the second discussion section.

Results and discussion section IV

The discussion section discusses some possible explanations of the findings but does not elaborate on the non-finding for custody vs. the finding on living. It would strengthen the paper if the authors could come up with an explanation for this as this is somewhat surprising for me as a reader.

Response: We have added a section that discusses the most obvious explanation for this; case severity.

Minor thing

A minor thing, but I hope that there is more recent work on unrealistic vignette stories than from 200 years ago.

Response: Our initial reference concerned the first notion of finding a story (even fictional stories) non-realistic. We have removed this reference.

Reviewer #2

While the paper focuses on an important topic, it would be good to further examine the impact of your findings. What does this mean for practitioners? What needs to happen in the field to reduce the negative impact on men?

Response: Thank you for this wise comment. We added a section "Implications for practitioners" where we discuss what practitioners need to do as well as a section "Implications for children and fathers". However, it is rather limited because we are cautious about letting this discussion end up as a scientific manifesto of men's rights in the family courts or delving into research on youth criminality because of the lack of fathers or father-figures.

Reviewer #3

Method

It is unclear from the manuscript whether the risk question was asked before the living recommendation.

Response: Thank you for pointing this out. We clarified this in the procedure section.

Results I

I recommend reporting and analyzing the risk data here.

Response: As much as we appreciate the interest in these data, we have decided against this recommendation for various reasons. We plan to write another paper based on this data. Perhaps unfair to the reviewer who is unable to see the data. Our strong suspicion, however, is that presentation of the risk data would risk the entire paper would be derailed into a paper on low levels of inter-rater reliability, and the potential societal issues such as limited rule of law that presents. We are cautious about letting the important gender discrimination data and message drown in the ocean of risk data. We hope that this argument is acceptable, and that the interested reviewer has patience for our future manuscript.

Results II

The sample size is very small: a total of 29 Swedish custody evaluators (8 men and 21 women). Relatedly, was there an interaction between the gender of the decision-maker and the gender of the parent? I am concerned that the sample size is too small to investigate this adequately.

Response: This is why we have statistics, also very small sample sizes can be significant if the effect size is large enough, or stated differently in this case that gender discrimination against men is sufficiently severe. In this context, that we do find significant findings in a small data set is an interesting finding in itself. We agree that the sample is small, and the present sample cannot detect any differences in ratings between the male and female participants. Since we cannot do this analysis, we have added this as a natural suggestion for future studies.

Discussion I

The authors acknowledge the risk of additional discrimination occurring in the judicial court. However, it could also be that courts "correct" the discriminatory decisions of evaluators. This should also be stated as a limitation.

Response: This is a good point. We have added a discussion on this as a limitation along with some empirical arguments that the courts do not correct the custody evaluations.

Reviewer #4

Overall, the manuscript indicates the exciting point of 'discrimination against men in custody dispute'. However, it requires more details on the significance of the study, its methodology and results for meaningful discussions. The manuscript's introduction still lacks an explanation of the importance of gender discrimination in men's studies.

Response: Thank you for this comment. We have revised the manuscript in and added more background

General point

It is still being determined whether the research is quantitative or qualitative.

Response: Thank you for this comment. To address this, we have changed the title to "Gender Discrimination in Swedish Family Courts: A Quantitative Vignette study".

Results

As the quantitative research, the sampling technique should be demonstrated. From which population can a representative samples of 29 social workers be resembled?

Response. This comment is confusing to us. First, a small sample by itself, does not mean that the representativeness of the sample must be limited. Furthermore, there is a limited number of custody evaluators in Sweden. We recruited participants from several municipalities in the middle of Sweden, including Stockholm, which is the largest city in Sweden. We have added this information in the Design and Participants section.

Results II

As for the qualitative research, the data results are invalid for hypothesis testing. The demographic data of all social workers should be included in discussions. Previous work on gender discrimination with the vignette method demonstrates that both the gender and social capital of participants play a significant role as well.

Response: This is a very good point. However, the social worker profession is heavily dominated by women, and our sample, although small, represents the gender distribution very well.

Discussion

Instead of discussion on the 'weaknesses' part of the study, the study should close the gap in its methodology and provide more insightful findings for further discussions.

Response: We have revised the discussion section by adding more topics such as implications for practitioners, and implications for fathers and children. We hope that this is an adequate revision.
