# Supplementary material for: Gender discrimination in Swedish family courts: A quantitative vignette study
Source: PLoS One. 2024 Aug 27;19(8):e0296210. doi: 10.1371/journal.pone.0296210 (PMC11349174; doi:10.1371/journal.pone.0296210)
Supplement: Attachment — Submitted filename: 2024-06-19 Rebutal letter Gender discrimination PLOS ONE.docx [file pone.0296210.s003.docx]

Dear editor/reviewer

We have made changes in PONE-D-23-40878R1 according to your suggestions as specified below.

Specifically, we suggest the following:

Section 'The primary care-taker preference', Paragraph 2

- This paragraph needs to be rewritten in order to A) ensure that provided statements are supported by recent references and B) clarify how athletics is associated with male gender stereotypes or is relevant to the discussion, or preferably, remove it altogether.

*Our response: We have removed the examples given in this paragraph. The paragraph now reads:*

*“Historically and traditionally, women have done these tasks more than men. This is particularly true when the child is an infant or very young. However, the importance of fathers becomes more pronounced as the child grows older, are more ready to be introduced to adulthood and how to handle the outside world. However, there is a strong notion that mothers are the primary caregiver in many cultures. As Warshak (1996) points out, there are several care-taking activities associated with traditional female gender stereotypes that are focused on in custody disputes. In contrast, such activities are typically not associated with male gender stereotypes. Similar to the tender years doctrine, the notion of the primary care-taker favored mothers over fathers.”*

Section, 'Significance of gender discrimination in child custody', Paragraph 1

- Rephrase what is meant by "This can only benefit women." (we have concerns about a potential statement of false equivalence being made here).

*Our response: This statement has been removed.*

Section, 'Method'

- Provide information on statistical analysis performed

*Our response: The missing inference test in the first paragraph of the results section has now been added.*

- Provide vignettes as SI file

*Out response: The Vignettes SI file labeled “NgaosuvanVignettes.pdf” has been attached to the submission*

- "In terms of custody recommendations, results showed no evidence for gender discrimination" - this is not in line with the results section above - the results were shown to be inconclusive for this part of the study - this needs to updated accordingly, as well as potential removal of the discussion regarding the floor effect.

*Our response: This has been removed: “ In terms of custody recommendations, results showed no evidence for gender discrimination. This fact that this variable was not significant is likely due to a floor effect in the data, where only six participants recommended sole custody. “*

Section, Implications for fathers and children, Paragraph 1

- we suggest that the discussion and comparison against the job market is not relevant and should be removed.

*Our response: This has been removed: “This is not true for discrimination in the job market, where a person discriminated against on a job application, can apply to other jobs. Fathers losing custody disputes have no such opportunity”.*

Reviewer #1: I believe that the added information about gender equality does not benefit the paper; the data on itself is interesting enough and does not need to be compared with gender bias against women. I would highly recommend to keep this paper separate from the comments about that gender equality for women is a disadvantage for men. It would strengthen both the introduction and discussion to focus just on this type of gender bias instead of minimizing other types in order to show the relevance of the research.

*Our response: We have deemphasized the gender equality comparisons in the manuscript.*

Reviewer #2: The authors have responded to feedback well and in doing so have made their publication much stronger. I applaud them for delving into such a difficult topic, and encourage them to do more work in the future and not to be too scared off by the women's rights movements. All genders should have equal rights and in an ideal world, one persons rights should not breach another persons rights.

*Our response: Thank you.*
